# Retraction: Restoration of Mal overcomes the defects of apoptosis in lung cancer cells

**DOI:** 10.1371/journal.pone.0296552

**Published:** 2023-12-28

**Authors:** The PLOS ONE Editors

After this article [1] was published, concerns were raised about some of the western blots in S2 Fig. Specifically, there appear to be similarities between multiple different areas in the backgrounds within the right Fig 2C panel in S2 Fig and within the left Fig 5I panel in S2 Fig. For some western blots in S2 Fig a full editorial assessment could not be completed due to the resolution of the images.

The nature of the issues observed in these images raises serious concerns regarding the reliability and integrity of the reported results and conclusions. Therefore, the *PLOS ONE* Editors retract this article.

PCY did not respond to the editorial decision. LTY, FM, HTZ, MZ, XHZ, and ZQL either did not respond directly or could not be reached.
